# Feasibility and beneficial effects of an early goal directed therapy after cardiac arrest: evaluation by conductance method

**DOI:** 10.1038/s41598-021-83925-3

**Published:** 2021-03-05

**Authors:** Ole Broch, Lars Hummitzsch, Jochen Renner, Patrick Meybohm, Martin Albrecht, Peter Rosenthal, Ann-Christine Rosenthal, Markus Steinfath, Berthold Bein, Matthias Gruenewald

**Affiliations:** 1 Department of Anesthesiology and Intensive Care Medicine, University Hospital Schleswig-Holstein, Campus Kiel, Kiel, Germany; 2 Department of Anesthesiology and Intensive Care Medicine, Elbe Hospital Stade, Stade, Germany; 3 Christian-Albrechts-University Kiel, Kiel, Germany; 4 Department of Anesthesiology and Intensive Care Medicine, Städtisches Krankenhaus Kiel, Kiel, Germany; 5 Department of Anesthesiology, Intensive Care, Emergency and Pain Medicine, University Hospital Würzburg, Würzburg, Germany; 6 Department of Anesthesiology and Intensive Care Medicine, Asklepios Hospital St. Georg, Hamburg, Germany

**Keywords:** Physiology, Medical research

## Abstract

Although beneficial effects of an early goal directed therapy (EGDT) after cardiac arrest and successful return of spontaneous circulation (ROSC) have been described, clinical implementation in this period seems rather difficult. The aim of the present study was to investigate the feasibility and the impact of EGDT on myocardial damage and function after cardiac resuscitation. A translational pig model which has been carefully adapted to the clinical setting was employed. After 8 min of cardiac arrest and successful ROSC, pigs were randomized to receive either EGDT (EGDT group) or therapy by random computer-controlled hemodynamic thresholds (noEGDT group). Therapeutic algorithms included blood gas analysis, conductance catheter method, thermodilution cardiac output and transesophageal echocardiography. Twenty-one animals achieved successful ROSC of which 13 pigs survived the whole experimental period and could be included into final analysis. cTnT and LDH concentrations were lower in the EGDT group without reaching statistical significance. Comparison of lactate concentrations between 1 and 8 h after ROSC exhibited a decrease to nearly baseline levels within the EGDT group (1 h vs 8 h: 7.9 vs. 1.7 mmol/l, P < 0.01), while in the noEGDT group lactate concentrations did not significantly decrease. The EGDT group revealed a higher initial need for fluids (*P* < 0.05) and less epinephrine administration (*P* < 0.05) post ROSC. Conductance method determined significant higher values for preload recruitable stroke work, ejection fraction and maximum rate of pressure change in the ventricle for the EGDT group. EGDT after cardiac arrest is associated with a significant decrease of lactate levels to nearly baseline and is able to improve systolic myocardial function. Although the results of our study suggest that implementation of an EGDT algorithm for post cardiac arrest care seems feasible, the impact and implementation of EGDT algorithms after cardiac arrest need to be further investigated.

## Introduction

In Europe, 150,000–400,000 people per year suffer out of hospital cardiac arrest^1^. The number of patients with return of spontaneous circulation (ROSC) varies between 27 and 61%^2,3^.

It must be noted, however, that initial ROSC alone is not decisive for patient’s survival. Beside successful cardiopulmonary resuscitation, establishment and maintenance of stable hemodynamic conditions are further important targets in the post ROSC time period. However, despite ROSC and intensive therapy, 50–70% of the patients die in the post-resuscitation period^4–6^. Factors like hyperoxaemia, hyperglycaemia, hypocapnia, hypotension and fever appear to have a significant negative effect on patient’s outcome^7–9^. To avoid mismatch of oxygen supply and consumption in the post-cardiac arrest period, hemodynamic stabilization by individually titrated volume and/or catecholamine therapy plays another important role. In this context, an early goal directed therapy (EGDT) seems to have potential beneficial impact on post-cardiac arrest syndrome^10,11^. However, most of the mentioned studies performed EGDT after cardiac arrest in association with a therapy bundle containing other therapeutic interventions. Moreover, post-resuscitation EGDT was compared to a historical control group which received none of these therapies. Recent studies indicate that early hemodynamic stabilization towards higher blood pressure values is associated with smaller myocardial damage and increasing survival rates after cardiac arrest^10,12–14^.

The aim of our prospective animal study was therefore to investigate the feasibility and the impact of an established EGDT algorithm on myocardial damage and function after cardiac resuscitation compared to a noEGDT group. We evaluated effects of EGDT by established markers of ischemia and myocardial function by advanced hemodynamic monitoring, including the conductance method. cTnT and lactate concentrations were defined as primary outcome parameters, while hemodynamics represented secondary outcome parameters.

We hypothesized that an EGDT based on variables like mean arterial pressure (MAP), central venous pressure (CVP), ejection fraction (EF) and central venous oxygen saturation (S_cv_O_2_) after cardiac arrest is in principle feasible and leads to an improved myocardial function.

## Methods

The study was approved by the local Animal Investigation Committee, Christian-Albrechts-University Kiel, Ministry for Agriculture, Environment and Rural Areas, Schleswig–Holstein (Permit Number: V 312-72241.121-39(3-1/10)). The present animal investigation was conducted in consideration of the National Institutes of Health guide for the care and use of Laboratory animals (NIH Publications No. 8023, revised 1978) and complied with the ARRIVE guidelines.

Thirty german domestic pigs with a weight between 28 and 34 kg were included. Preparation of the animals and experimental setting were based on the protocols described previously^15–17^. Briefly, after fasting overnight with free access to water, the animals were premedicated with the neuropleptic azaperone (4 mg/kg), ketamine (1.0 mg/kg) and atropine (10 µg/kg) 1 h before surgery. Thereafter, propofol (2 mg/kg) and sufentanil (0.5 µg/kg) were administered through a venous access, typically placed in an ear vein, followed by endotracheal intubation. General anaesthesia was performed with the aim of avoiding pain and minimizing distress or suffering for the animals. Pigs were ventilated with the Sulla 808-V ventilator (Dräger AG, Lübeck, Germany) in a volume-controlled mode with a tidal volume of 8 ml/kg, a positive end-expiratory pressure of 5 cm H_2_O, an I:E ratio of 1:1.5 and a FiO_2_ of 0.4. Respiratory rate was adjusted to achieve normocapnia (pCO_2_ 35–40 mmHg). A pulse oximeter was placed on the ear to monitor oxygen saturation (M-CaiOV, Datex-Ohmeda, Helsinki, Finland) and cardiac rhythm was monitored by standard 5-channel electrocardiogram. Anesthesia was maintained by continuous infusion of propofol (4–8 mg/kg/h) and sufentanil (0.3 µg/kg/h). We repeatedly performed pain stimuli like tail clamping and focused on the corneal reflex and lacrimation to detect an inadequate depth of anaesthesia. If necessary, additional sufentanil and propofol was injected. To maintain temperature between 37.0 and 38.0 °C a heating blanket was used. During the whole study period, the pigs received an infusion of cristalloid solution (Sterofundin, B. Braun-Melsungen AG, Germany) with a flow rate of 10 ml/kg/h and an antibiotic prophylaxis with cefuroxime.

Under sterile conditions, a 7 Fr triple lumen central venous catheter (Arrow International, Inc. Reading, PA, USA) was inserted percutaneously in the left internal jugular vein. Thereafter, a 7.5 Fr introducer was placed in the right jugular vein for insertion of a temporary catheter for electrical induction of ventricular fibrillation. Subsequently, a 4.0 Fr thermistor tipped catheter for thermodilution and pulse contour analysis was inserted percutaneously into the femoral artery (PV 2015L20, Pulsiocath, Pulsion Medical Systems AG, Munich, Germany). Additionally, surgical preparation of the right femoral vein was carried out and a 14 Fr introducer for insertion of an occlusion catheter (Fogarty, 120806F Edwards Lifescience, Irvine, CA) was placed into the vein. The right carotid artery was also surgically uncovered for placement of an 8 Fr introducer followed by a 7 Fr conductance catheter (CD Leycom, CA-71103-PL, Hengelo, The Netherlands). A transesophageal echocardiography probe was firstly inserted to control the position of the conductance catheter and, secondly, for measurement of EF by the simpson’s method of discs.

After connection to the PiCCOplus Monitoring system (Version 6.0, Pulsion Medical Systems AG, Munich, Germany), this system allows discontinuous measurement of CO_TPTD_ by transpulmonary thermodilution and other hemodynamic variables, measurement of CVP, S_cv_O_2_, transpulmonary thermodilution cardiac output (CO_TPTD_) and stroke volume (SV_TPTD_) was derived from central venous catheter and PiCCO catheter, respectively. For assessment of CO_TPTD_ and SV_TPTD_, thermodilution measurements were obtained by injecting 10 ml ice cold saline (≤ 8 °C) through the central venous catheter. Without taking account of the respiratory cycle, injections were performed at least three times. In case of a difference of CO_TPTD_ ≥ 15%, preceding measurement was discarded and calibration repeated. Transesophageal echocardiography for evaluation of EF was performed by a single experienced examiner using a multiplane transesophageal echocardiography probe (GE Healthcare 6 T TEE probe, Vivid i-System, Wauwatosa USA). Calculations by the conductance catheter method (CD Leycom Sigma, Zoetermeer, The Netherlands) included left ventricular end-systolic elastance (Ees), preload recruitable stroke work (PRSW, stroke work-to-end-diastolic volume relationship), diastolic compliance (EDPVR, end-diastolic pressure–volume relation) and a parameter of early diastolic relaxation (Tau). For determination of preload-independent parameters during the measurements by conductance method, an occlusion catheter was placed in the inferior vena cava and inflated for 5–10 s.

**Figure 1:**
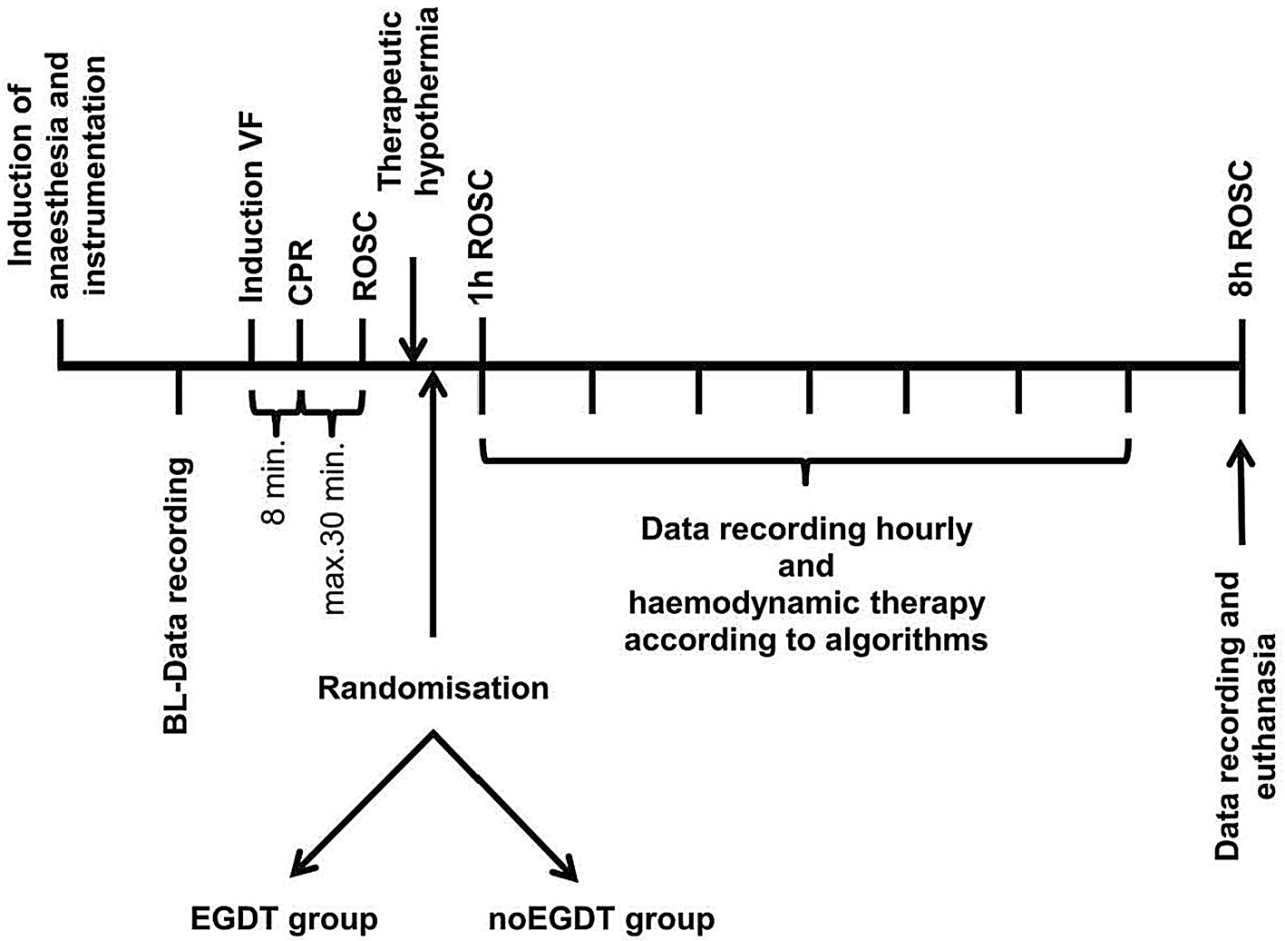
Illustration of experimental protocol. Data collection was performed at baseline and hourly after ROSC. *BL* baseline, *VF* ventricular fibrillation, *CPR* cardiopulmonary resuscitation, *ROSC* return of spontaneous circulation, *EGDT* early goal directed therapy.

After instrumentation, all respiratory, hemodynamic and echocardiographic data recordings were performed at baseline. Arterial and central venous blood samples were collected for blood gas analysis (GEM Primer 4000, Werfen Germany, Munich). All measurements were performed with the animal in supine position. The experimental protocol is illustrated in Fig. 1.

**Figure 2:**
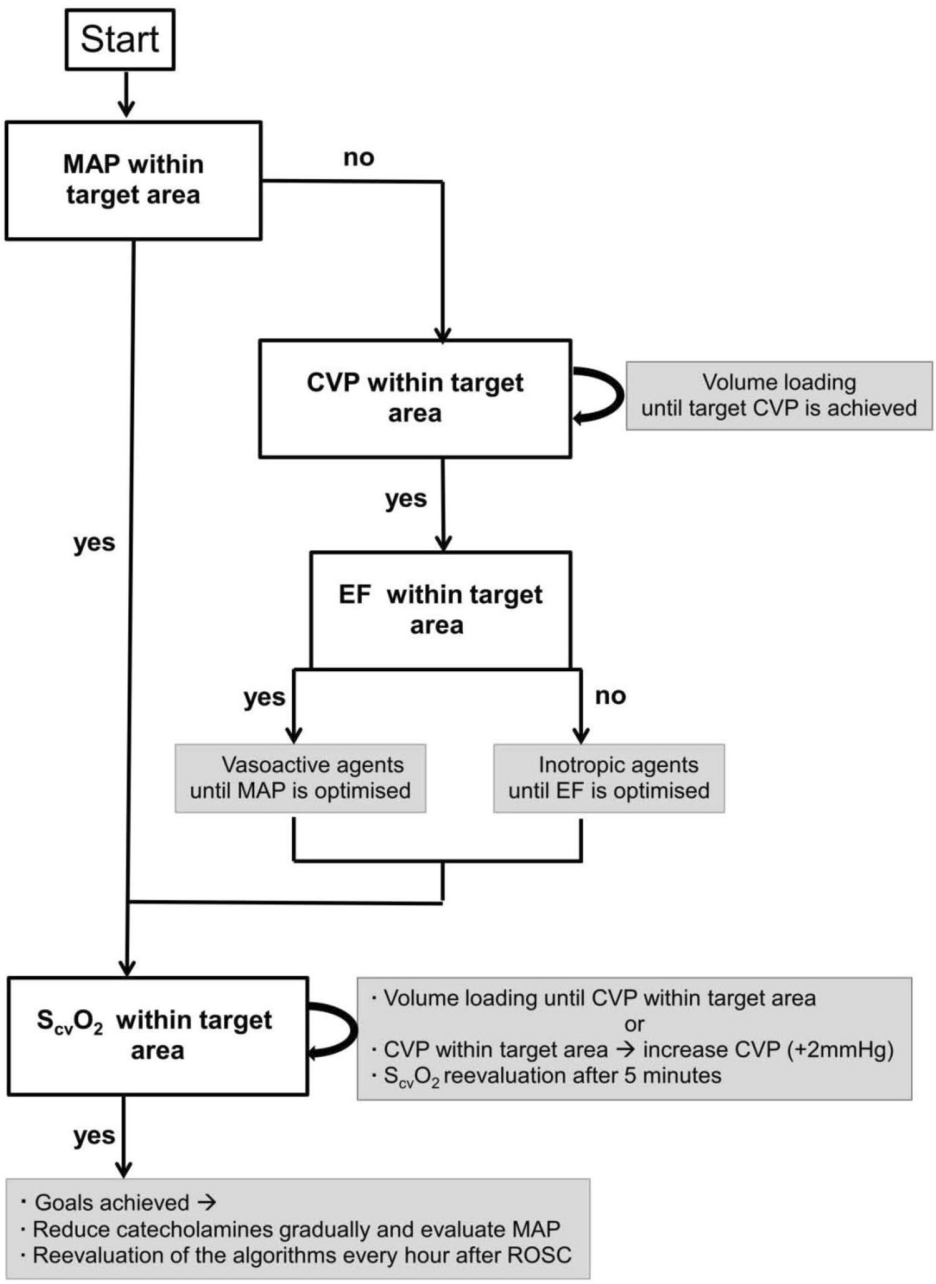
Therapy algorithm.

To prevent any blood clotting caused by the placed catheters, pigs received 100 IE/kg heparin. Thereafter, a temporary catheter for electrical induction of ventricular fibrillation was advanced through the introducer in the right jugular vein. After induction of ventricular fibrillation, mechanical ventilation was stopped and cardiopulmonary resuscitation was started after 8 min of cardiac arrest in accordance to the guidelines from the European resuscitation council^18^. For the first 4 min basic life support was carried out with a chest compression of 100/min and a relationship of compression-to-ventilation of 30:2. Advanced cardiac life support was initiated by one biphasic defibrillation using 4 J/kg (Corpuls 3 (ED 530), Kaufering, Germany). Besides continuous chest compression further ventilation was performed with 100% oxygen and an initial respiratory rate of 20/min. All pigs received 5 mg/kg amiodarone and intermittent every 2 min 15 µg/kg epinephrine or 0.4 IE/kg vasopressin. Cardiopulmonary resuscitation was stopped if ROSC was observed which was defined as maintenance of an unassisted pulse and a systolic aortic blood pressure of ≥ 60 mmHg lasting for ten consecutive minutes according to the Utstein-style guidelines^19^. If resuscitation remained unsuccessful after 30 min, cardiopulmonary resuscitation was terminated. After ROSC, a mild therapeutic hypothermia was initiated in all animals to achieve a temperature ranging between 32 and 34 °C and inspiratory oxygen concentration was reduced. Just before the end of the first hour after ROSC, animals were randomized either into EGDT or noEGDT group. Subsequently, measurements by conductance method, transpulmonary thermodilution via PiCCO catheter and transesophageal echocardiography were performed every hour after ROSC. Additionally, arterial and central venous blood gas analyses were carried out. Blood samples for quantification of cardiac troponin T (cTnT), lactate and lactate dehydrogenase (LDH) were obtained at various time points after ROSC. After completion of the measurements, a therapy algorithm was established and hemodynamic therapy was conducted in dependence of group allocation. The core variables, MAP, CVP, EF and S_cv_O_2_ were evaluated in a fixed and predefined order. Therapeutic options were limited to a targeted repetitive volume administration (bolus of 250 ml balanced crystalloids) and the targeted adjustment (stepwise 1–10 µg/min) of vasopressors (norepinephrine) or inotropes (epinephrine). Hemodynamic therapy and target variables were similar but threshold values differed between both groups. In the EGDT group, target area was MAP > 80% of the baseline, CVP > 7 mmHg, EF > 60% and S_cv_O_2_ > 70%. In the noEGDT group, thresholds of target variables were defined by a stochastic random model on an hourly basis. Thresholds could be equal, higher or lower compared to the EGDT group. Limit ranges in the noEGDT group were defined as MAP 55–120% of the baseline, CVP 2–8 mmHg, EF 40–70% and S_cv_O_2_ 50–80% (Supplement 1). The detailed therapy algorithm is displayed in Fig. 2. For both groups intervention thresholds indicating direct vital threat were defined as drop of < 40% of baseline for MAP and < 45% for S_cv_O_2_. If values fell below the set threshold, individual therapeutic measures were initiated by an experienced physician independently from the therapy algorithm.

Therapy was stopped if pre-defined targets were achieved or at the start of a new period of measurements. Latest therapeutic algorithm was initiated after seven hours and ended after data recording eight hours after ROSC. After completion of the trial period, animals were euthanized by an overdose of sufentanil, propofol and potassium chloride.

### Statistical analysis

Statistical comparisons were performed using commercially available statistics software (GraphPad Prism 6, Version 6.03, GraphPad Software Inc., San Diego, CA, USA). To determine the distribution between the population and the variables studied, a Shapiro–Wilk test was performed. Pooled data of both groups were compared by unpaired t-test (normally distributed) or Mann–Whitney-U-Test (not normally distributed). In case of normally distributed data, two-way ANOVA was performed for analysis of repeated measurements over the period of time. Occasional missing data in the time kinetics were filled by the “multiple imputation technique” (MIT) which is available in the STATA 13 statistics software (StataCorp LP, CollegeStation, TX, USA). To evaluate the effect of hemodynamic therapy, variables at one and eight hours after ROSC were compared by paired t-test or Wilcoxon sign rank test. All variables are expressed as mean ± SD or median [25–75% quartiles].

### Ethics approval

The study was approved by the local Animal Investigation Committee, Christian-Albrechts-University Kiel, Ministry for Agriculture, Environment and Rural Areas, Schleswig–Holstein (Permit Number: V 312-72241.121-39(3-1/10)). The present animal investigation was conducted in consideration of the National Institutes of Health guide for the care and use of Laboratory animals (NIH Publications No. 8023, revised 1978) and complied with the ARRIVE guidelines.

## Results

A total of 30 pigs were investigated from which 21 achieved ROSC after 8 min of cardiac arrest. Two animals died within the first hour after ROSC due to intra-abdominal bleedings, so that 19 pigs were randomly assigned to each group (EGDT group, N = 10; noEGDT group, N = 9). Subsequently, three animals of each group died, so that seven animals in the EGDT group and six animals in the noEGDT group could be included into the final analysis. One animal in the EGDT group showed already marked increase in cTNT levels at baseline. Descriptive and resuscitation data are presented in Table 1. Note that the standard deviation but not the mean of the duration of cardiopulmonary resuscitation differed notably between both groups and was shifted towards shorter times in the control group (noEGDT).

**Table 1:** Descriptive data.

	EGDT (N = 7)	noEGDT (N = 6)	P-value
Weight (kg)	32 ± 2.4	32 ± 2.7	0.76
Duration CPR (min)	10 ± 3	10 ± 8	0.38
Cumulative energy (J)	656 ± 328	667 ± 621	0.70
Epinephrine during CPR (µg)	1350 ± 645	828 ± 497	0.18
Amiodarone during CPR (mg)	150 ± 43	171 ± 104	0.85
Vasopressin during CPR (IE)	17 ± 10	18 ± 8	0.99

Values are presented as mean ± SD.

*CPR* cardiopulmonary resuscitation.

Hemodynamic and laboratory variables at baseline are shown in Table 2. There were no significant differences between both groups.

**Table 2:** Hemodynamic variables and laboratory parameters at baseline.

	EGDT (N = 7)	noEGDT (N = 6)	P-value
HR (bpm)	100 ± 19	101 ± 19	0.86
MAP (mmHg)	86 ± 19	99 ± 15	0.22
CVP (mmHg)	4.0 ± 3.0	4.0 ± 2.0	0.60
CO_TPTD_ (l/min)	4.2 ± 0.7	4.6 ± 0.8	0.35
EF (%)	67 ± 10	62 ± 6	0.29
SV^c^ (ml)	59 ± 18	64 ± 13	0.65
Ees^c^ (mmHg/µl)	1.9 ± 1.1	1.5 ± 1.4	0.42
PRSW^c^ (mmHg)	76 ± 20	64 ± 22	0.42
EDPVR^c^ (mmHg/µl)	0.3 ± 0.1	0.3 ± 0.1	0.75
Tau^c^ (ms)	40 ± 26	24 ± 9	0.27
pH	7.5 ± 0.1	7.5 ± 0.0	0.28
S_cv_O_2_ (%)	78 ± 8	72 ± 9	0.23
Lactate (mm/l)	1.4 ± 0.8	1.6 ± 0.8	0.71
cTNT (pg/ml)	47 ± 91	3.0 ± 1.0	0.15

Values are presented as mean ± SD.

*HR* heart rate, *MAP* mean arterial pressure, *CVP* central venous pressure, *CO*_*TPTD*_ cardiac output derived from transpulmonary thermodilution, *EF* ejection fraction, *SV* stroke volume, *Ees* end-systolic elastance, *PRSW* preload recruitable stroke work, *EDPVR* end-diastolic pressure–volume relation, *Tau* early diastolic relaxation, *S*_*CV*_*O*_*2*_ central venous oxygen saturation, *cTNT* cardiac troponin T, ^*c*^ variables measured by conductance catheter.

The hourly control of the target variables led to the implementation of the therapeutic algorithm (MAP and/or S_CV_O_2_ not in target range) in total 47 times in the EGDT group (average of 6.7 per animal) and 42 times (average of 7 per animal) in the noEGDT group. The hemodynamic algorithm cycles were more often successfully completed within EGDT group (83% of attempts) compared to noEGDT group (71% of attempts). In the EGDT group, targeted values could be reached in 42 of 47 therapeutic algorithms (85%) and in 31 of 42 algorithms for the noEGDT group (74%). Therapeutic algorithm required 18 min (± 14) in the EGDT group and 19 min (± 17) in the noEGDT group by average. In the noEGDT group, MAP fell twice considerably below life-threatening threshold values and needed further rescue intervention. An overview of cardiac variables and S_cv_O_2_ for EGDT and noEGDT group at different time points is shown in Table 3. Pooled data revealed significant differences for EF, PRSW, EDV and dp/dt_max_ between animals in the EGDT and noEGDT group. Statistically significant differences between the EGDT and the noEGDT group were also observed for the following parameters and time points: PRSW (8 h after ROSC, P < 0.01), EDPVR (4 h after ROSC, P < 0.05), EDV (8 h after ROSC, P < 0.01) and Dp/dt_max_ (4 h after ROSC, P < 0.05; Table 3).

**Table 3:** Hemodynamic variables and parameters of myocardial function during the study period.

	BL		1 h ROSC		4 h ROSC		8 h ROSC		Pooled data	
	EGDT	No EGDT	EGDT	No EGDT	EGDT	No EGDT	EGDT	No EGDT	EGDT	No EGDT
MAP (mmHg)	86 ± 19	99 ± 15	71 ± 13	77 ± 12	79 ± 27	80 ± 8	89 ± 34	76 ± 14	79 ± 7	80 ± 7
SV^c^ (ml)	59 ± 18	64 ± 13	47 ± 11	49 ± 19	42 ± 14	45 ± 19	38 ± 8	50 ± 13	45 ± 6	49 ± 6
EF (%)	67 ± 10	62 ± 6	62 ± 11	56 ± 10	58 ± 7	43 ± 17	52 ± 6	46 ± 15	57 ± 5	51 ± 7*
PRSW^c^ (mmHg)	76 ± 20	64 ± 22	55 ± 17	52 ± 6	57 ± 8	50 ± 6	68 ± 20	**37 ± 12**^**##**^	64 ± 8	49 ± 7*
Ees^c^ (mmHg/ml)	1.9 ± 1.0	1.5 ± 1.4	0.8 ± 0.2	1.1 ± 0.6	1.5 ± 0.7	1.4 ± 0.7	1.6 ± 1.1	0.8 ± 0.5	1.5 ± 0.3	1.3 ± 0.3
EDPVR^c^ (mmHg)	0.3 ± 0.1	0.3 ± 0.1	0.3 ± 0.1	0.3 ± 0.1	0.3 ± 0.1	**0.5 ± 0.2**^**#**^	0.4 ± 0.1	0.5 ± 0.2	0.3 ± 0.0	0.4 ± 0.1
EDV^c^ (ml)	78 ± 21	102 ± 18	71 ± 22	85 ± 24	77 ± 23	92 ± 33	69 ± 29	**113 ± 24**^**##**^	73 ± 10	99 ± 14*
EDP^c^ (mmHg)	21 ± 16	14 ± 5	18 ± 14	12 ± 11	9 ± 13	27 ± 22	18 ± 4	21 ± 7	16 ± 6	22 ± 7
Dp/dt_max_^c^ (mmHg/s)	2866 ± 1208	2445 ± 531	4054 ± 545	3611 ± 662	5083 ± 1265	**2912 ± 1231**^**#**^	3991 ± 2746	2052 ± 786	4452 ± 751	2879 ± 644*
Dp/dt_min_^c^ (mmHg/s)	− 1869 ± 992	− 2734 ± 826	− 1557 ± 335	− 1909 ± 367	− 1890 ± 647	− 1755 ± 1011	− 1964 ± 947	− 2014 ± 472	− 1845 ± 181	− 2033 ± 280
Tau^c^ (ms)	40 ± 26	24 ± 9	34 ± 19	22 ± 4	32 ± 27	37 ± 20	27 ± 11	26 ± 6	31 ± 5	28 ± 5
S_CV_O_2_ (%)	78 ± 8	72 ± 9	80 ± 9	82 ± 9	77 ± 7	67 ± 12	72 ± 13	59 ± 12	75 ± 5	70 ± 7
CO_TPTD_ (l/min)	4.2 ± 0.7	4.6 ± 0.8	4.6 ± 1.9	4.8 ± 1.7	4.3 ± 1.8	4.0 ± 1.6	4.8 ± 0.7	4.5 ± 1.2	4.5 ± 1.4	4.5 ± 1.3
SSVRI (dyn*s*cm^−5^*m^2^)	1766 ± 376	1656 ± 608	1350 ± 724	1308 ± 539	1572 ± 965	1737 ± 716	1393 ± 739	1308± 359	1505 ± 726	1500 ± 555

Values are presented as mean ± SD.

*MAP* mean arterial pressure, *SV* stroke volume, *EF* ejection fraction, *PRSW* preload recruitable stroke work, *Ees* end-systolic elastance, *EDPVR* end-diastolic pressure–volume relation, *EDV* end-diastolic volume, *EDP* end-diastolic pressure, *Dp/dt*_*max*_ maximal rate of rise of left ventricular pressure, *Dp/dt*_*min*_ minimal rate of rise of left ventricular pressure, *Tau* early diastolic relaxation, *S*_*CV*_*O*_*2*_ central venous oxygen saturation, *CO*_*TPTD*_ cardiac output derived from transpulmonary thermodilution, *SVRI* systemic vascular resistance index, ^*c*^ variables measured by conductance catheter.

^#^P < 0.05; ^##^P < 0.01 (repeated measures two-way ANOVA); *P < 0.05 (unpaired t-test or Mann–Whitney-U-Test).

**Figure 3:**
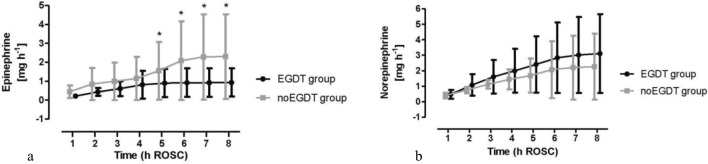
Cumulative consumption of epinephrine (**a**) and norepinephrine (**b**). *ROSC* return of spontaneous circulation. Bars denote SD.

The total amount of epinephrine was significantly lower in the EGDT group (Fig. 3a), whereas norepinephrine was used less frequently in the noEGDT group without reaching statistical significance (Fig. 3b).

**Figure 4:**
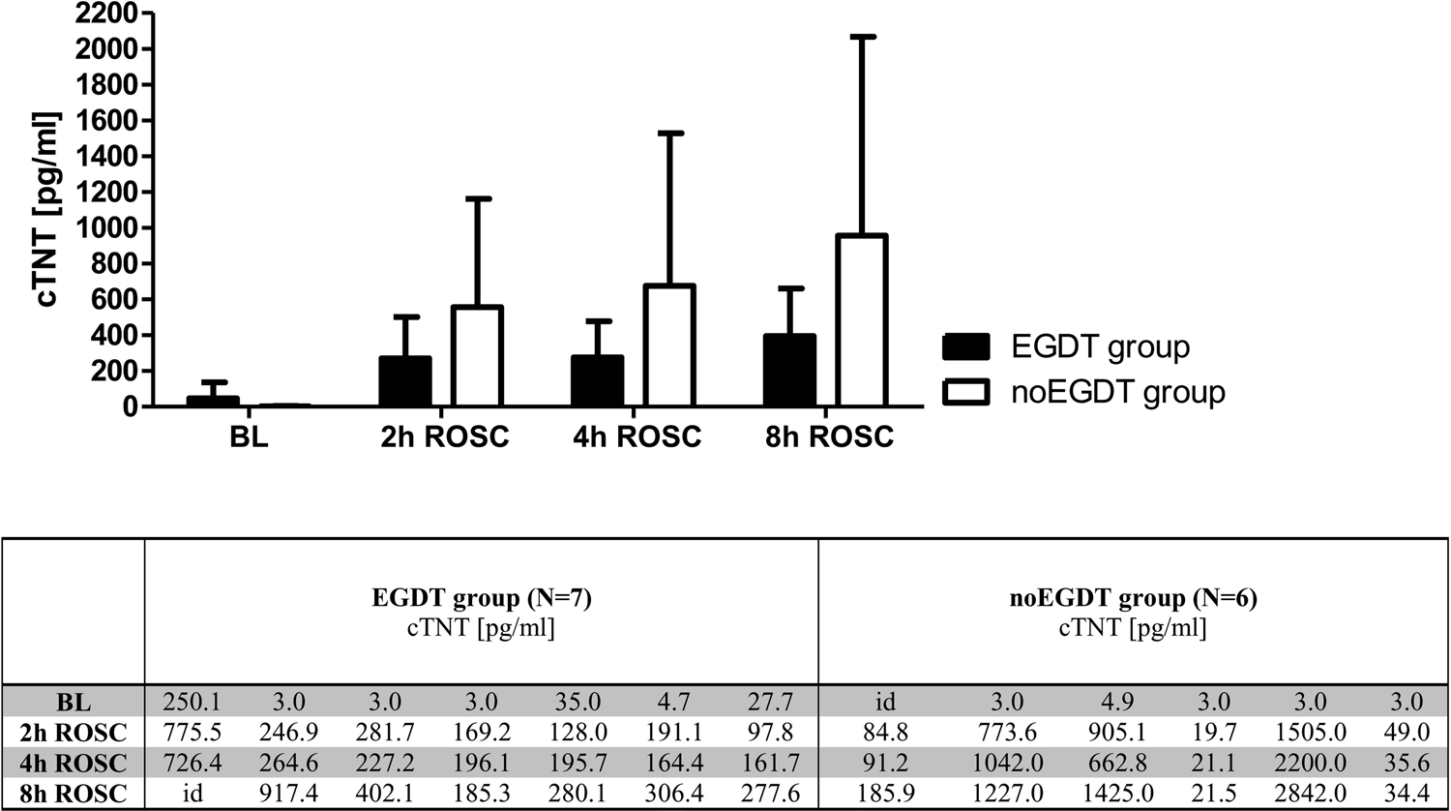
Mean concentrations of plasma cTNT at baseline, 2 h, 4 h and 8 h after ROSC in the EGDT and noEGDT group (upper panel). Columns show the mean, bars denote SD. cTNT values for each animal are depicted in the table (lower panel). *Id* invalid data.

Cardiac TnT levels did not show statistically significant differences between the EGDT and noEGDT group (Fig. 4).

**Figure 5:**
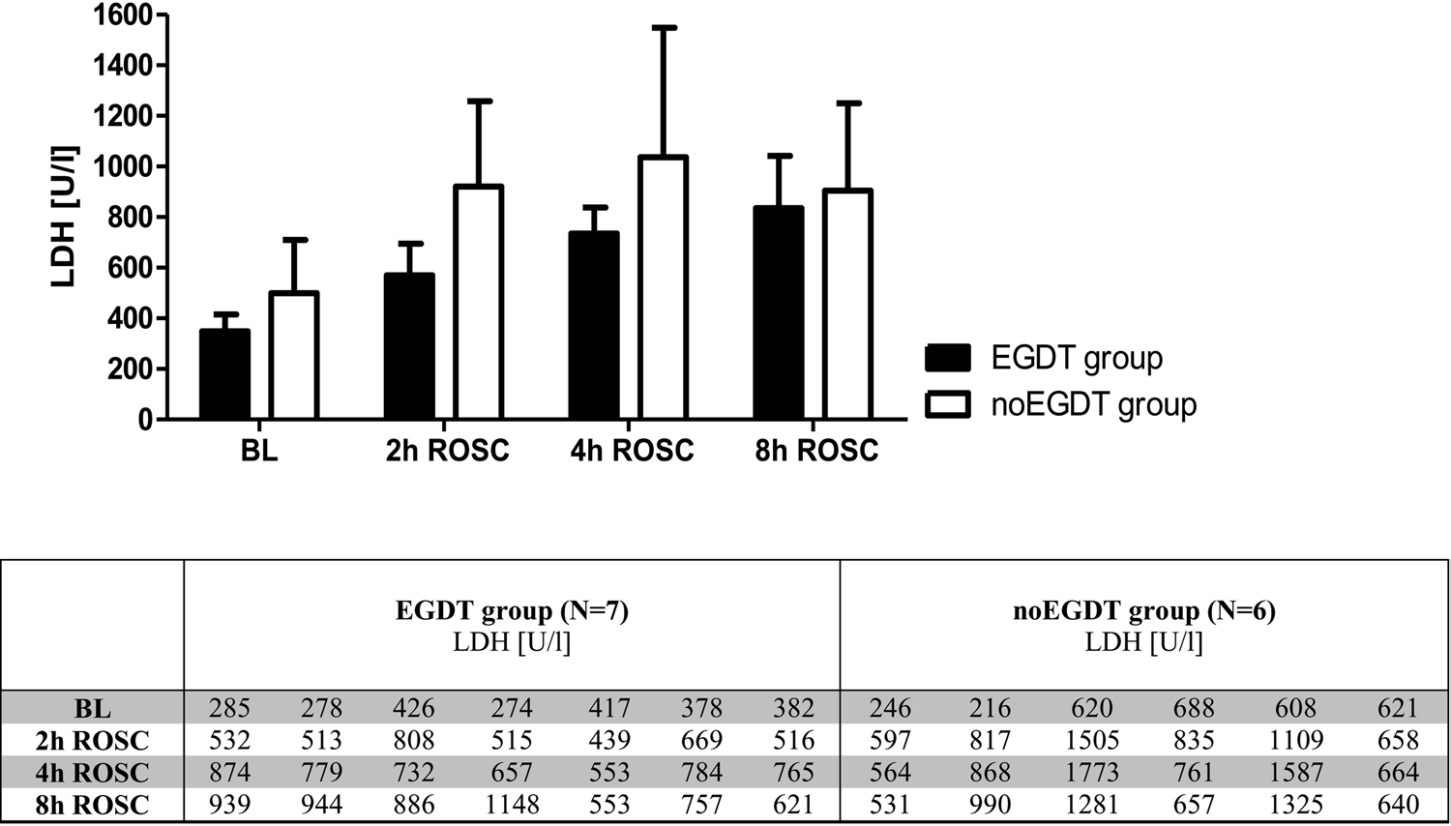
Mean catalytic activities of plasma LDH at baseline, 2 h, 4 h and 8 h after ROSC in the EGDT and noEGDT group (upper panel). Colunms show the mean, bars denote SD. Catalytic LDH activities for each animal are depicted in the table (lower panel).

LDH at baseline, 2 h, 4 h and 8 h after ROSC showed no significant differences between both groups (Fig. 5).

**Figure 6:**
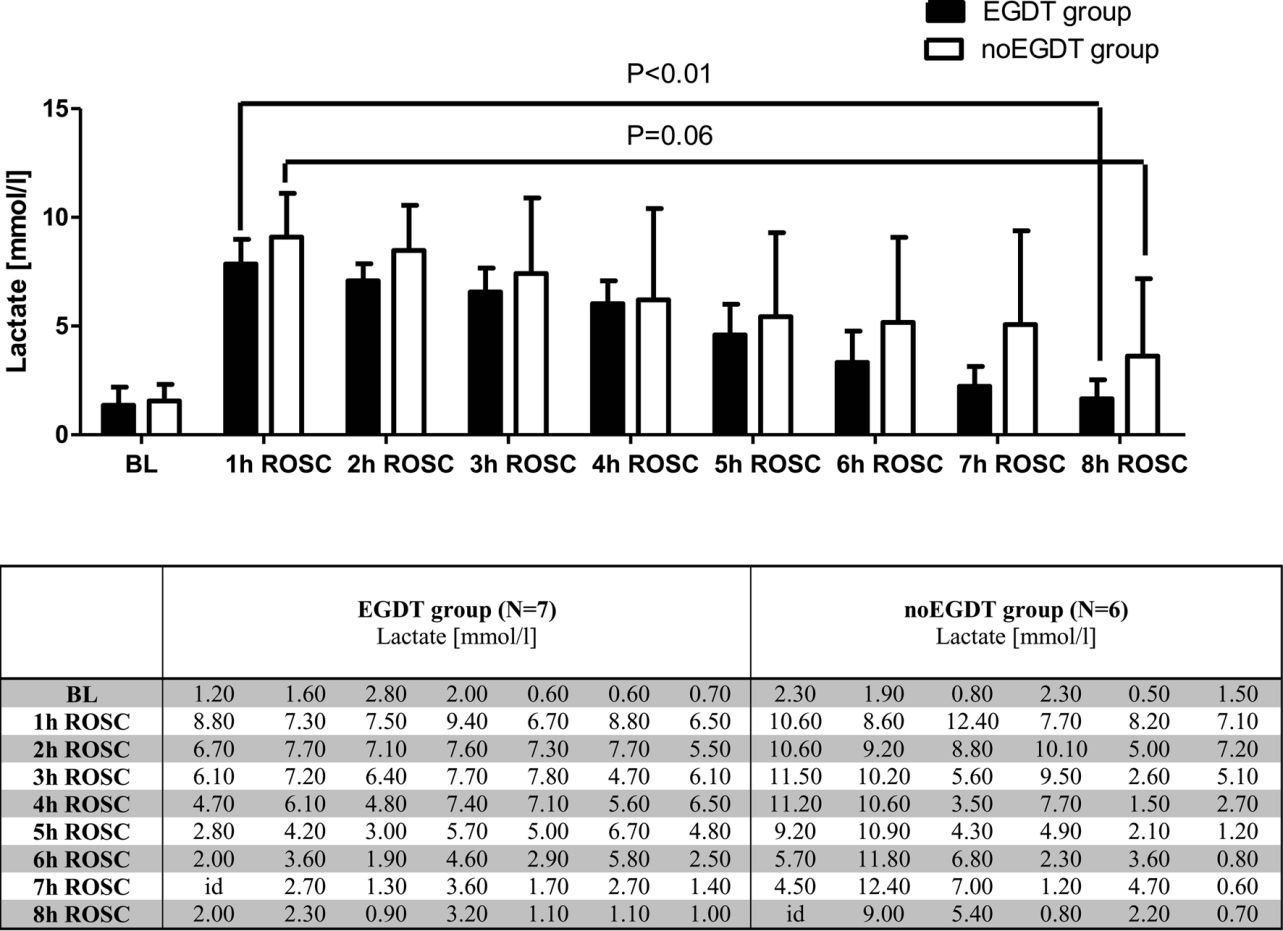
Mean concentrations of plasma lactate at baseline and between 1 h and 8 h after ROSC in the EGDT and noEGDT group (upper panel). Columns show the mean, bars denote SD. Lactate values for each animal are depicted in the table (lower panel). *id* invalid data.

Immediately after CPR, lactate concentrations increased up to 7–10 mmol/l in both groups. Comparison of lactate concentrations between 1 and 8 h exhibited a significant decrease to nearly baseline levels in the EGDT group (1 h vs 8 h ROSC: 7.9 vs. 1.7 mmol/l, *P* < 0.01), while in the noEGDT group lactate concentrations did not decrease significantly (1 h vs. 8 h ROSC: 9.1 vs. 3.8 mmol/l, *P* = 0.06; Fig. 6).

Total amount of fluids per therapeutic cycle displayed no significant difference between the two groups (EGDT 9.8 ± 11.1 ml/kg, noEGDT 7.6 ± 10.4 ml/kg, *P* = 0.259; Supplement 2). With respect to target fluid administration, we recorded higher fluid administration in the initial 2 h after ROSC in EGDT animals (Supplement 2). Subanalysis of therapeutic cycles within the EGDT group demonstrated a significant higher volume loading compared to the noEGDT if the targets have been achieved (EGDT: 9.1 ± 11.6 ml/kg vs noEGDT: 3.6 ± 6.9 ml/kg, *P* = 0.026; not shown).

## Discussion

By using a randomized animal model, we could demonstrate that implementation of an EGDT algorithm after cardiac arrest and successful ROSC was in principle feasible. Furthermore, EGDT was associated with a more pronounced decrease of lactate concentrations to almost baseline levels and a significant improved systolic myocardial function, which was not observed in the noEGDT group. The EGDT group revealed significant reduced need for epinephrine and the absence of hemodynamic life-threatening situations requiring immediate therapeutic intervention.

Although 50% of initial successfully resuscitated patients die after administration to a hospital, there exist no evidence based guidelines for treatment of this specific group of patients^4,5^. A large proportion of deaths after cardiac arrest are caused by neurologic injury^20^. Therapeutic recommendations include percutaneous coronary intervention, target temperature management and goal directed hemodynamic optimization. However, performance and implementation of hemodynamic therapy after successful resuscitation is still elusive^21^. There are only few recommendations regarding hemodynamic post resuscitation care on which clinicians can orientate^6,11,22,23^. Rivers and colleagues have demonstrated that an early goal directed hemodynamic optimization could reduce mortality in septic patients^24^. In this context, a clinical study could demonstrate similarities in inflammatory response between septic patients and patients after cardiac arrest and successful ROSC^25^. Accordingly, other studies investigated the effect of an early goal directed hemodynamic optimization in patients after successful resuscitation^10,11,26^. However, the relevance of these studies is weak due to implementation of EGDT within a therapeutic bundle.

### Cardiac function

Because a potential beneficial impact of EGDT on cardiac function and myocardial damage after cardiac arrest was not studied systematically up to now, the aim of our study was to investigate these issues in an animal model.

We could demonstrate a positive impact of EGDT on systolic cardiac function in pigs after cardiac arrest and successful resuscitation. Thus, for example, PRSW as a pre- and afterload independent variable, EF and dp/dt_max_ as load-dependent parameters revealed significant higher values in the EGDT group compared to the noEGDT group. These observations were supported by the course of S_cv_O_2_. This parameter is used to evaluate the ratio of oxygen supply and demand and also serves as an indirect marker for myocardial function. In our study, S_cv_O_2_ in the EGDT group revealed a stable trend and no significant decrease over the time in contrast to the noEGDT group.

However, Ees as a parameter for cardiac contractility independent from pre- and afterload, exhibited no significant differences between both groups. Interestingly, after a considerable reduction caused by cardiac arrest, Ees demonstrated a continuous increase over time with reaching similar values at 8 h compared to baseline in the EGDT group. These findings were in accordance with other studies dealing with myocardial function after cardiac arrest. For example, Gazmuri et al. as one of a few investigated myocardial contractility in a cardiac arrest animal model by using a conductance catheter. The authors also observed a significant reduction in Ees^27^. Interestingly, in our study absolute values for EF were considerably higher compared to other investigations^27,28^. This could be explained by the absence of any early goal directed hemodynamic optimization in the aforementioned studies.

EGDT may solely be associated with improved myocardial function and hemodynamics^29,30^. For the assessment of diastolic myocardial function, we compared end-diastolic volume (EDV), Tau, dp/dt_min_, EDPVR and end-diastolic pressure (EDP). Except for EDV, there were no significant differences between both groups. There are rare data concerning cardiac diastolic function after cardiac arrest. Kern and colleagues obtained an increased left ventricular end diastolic pressure (LVEDP) and Tau in domestic pigs following cardiac arrest^28^. In this context, another study dealing with survivors of out-of-hospital cardiac arrest reported an increased LVEDP in hemodynamic instable patients compared to the stable group^31^.

### Hemodynamics and laboratory parameters

With respect to hemodynamic stabilization, recent studies recommended the usage of catecholamines in the post resuscitation period^6,23,32^. In daily clinical routine, MAP, lactate and urine output are often used for control of catecholamine therapy. Accordingly, low demand of cardiovascular drugs, especially epinephrine, seem to have positive impact on patient’s outcome^33,34^.

It must be emphasized, however, that in our study the EGDT group exhibited significant lower doses of epinephrine. This might be explained by the differentiated therapy with volume and catecholamines in context of an early goal directed hemodynamic optimization. Several studies have shown, that despite of total amount of fluids, timing of fluid loading seems to play a more important role in hemodynamic optimization resulting in significant higher values for CO and MAP^30,35^. In this context, recent studies could demonstrate, that a target range of MAP between 80 and 100 mmHg after cardiac arrest resulted in less myocardial injury. Furthermore, severe neurological dysfunction was associated with MAP thresholds < 75 mmHg^13,36–38^.

With respect to myocardial damage after cardiac arrest, we could demonstrate that an early goal directed hemodynamic optimization in the post resuscitation period was associated with lower values for cTnT and LDH without reaching statistical significance. There are several causes associated with a troponin increase after cardiac arrest. One of the most common reasons is myocardial ischemia due to coronary artery stenosis or occlusion^39^. However, as we investigated young and healthy animals and ventricular fibrillation was electrically induced, coronary lesions appears unlikely. Other factors with influence on troponin release after cardiac arrest is the time required to reach ROSC and numbers of defibrillations^40,41^. With respect to our study, these two factors were negligible as we observed no significant differences between EGDT and noEGDT group. In this context, it must be noted that the differing standard deviations of duration of cardiopulmonary resuscitation in both groups were probably based on randomness and the small sample size. However, when looking at the distribution of the individual animals, it is noticeable that the distribution of cardiopulmonary resuscitation period in the noEGDT group was shifted towards shorter times. Therefore, the effect on myocardial ischemia cTNT may even be underestimated.

In our study, we observed the highest levels for lactate one hour after ROSC in both groups. There was a continuous decrease of lactate in both groups over the time, while only the EGDT group achieved similar concentrations after eight hours of ROSC compared to baseline. Elevation of serum lactate is a surrogate parameter of inadequate tissue perfusion and is accompanied with increased mortality and poor neurological outcome after cardiac arrest^42,43^. Several studies demonstrated a relationship between epinephrine and increased lactate levels in patients with septic shock^44,45^. As we observed significant lower total amounts of epinephrine in the EGDT group, this might have influenced our results for lactate concentrations.

Although there is a large proportion of deaths after cardiac arrest due to neurologic injury^20^, we did not measure biomarkers of neurologic injury in the present study due to methodological issues (partial occlusion of the carotid artery for conductance measurement). Therefore, we were unable to make a statement on the impact of hemodynamic optimization on neurologic injury after cardiac arrest. In this context, some investigations emphasized the use of regional cerebral oximetry or EEG derived variables during cardiopulmonary resuscitation and post-resuscitation period^46,47^. Early goal directed hemodynamic optimization after cardiac arrest seems capable of improving cerebral oxygenation. However, the extent of the influence on brain damage and neurological outcome remains elusive^48^.

As suggested by Gaieski and colleagues, the EGDT algorithm should be compared with therapies typically used in daily clinical routine^14^. In this context, it must be emphasized that there exist no concrete standards for post cardiac arrest care^49^. This seems to be one reason for the observed different mortality rates in various hospitals and regions indicating on different therapy approaches^50^. Therefore, in order to simulate these issues we used a dynamic concept for the noEGDT group.

### Limitations

There are some limitations of our study that should be addressed. As we investigated healthy pigs with normal cardiac and pulmonary function, our results cannot directly transferred to patients after cardiac arrest exhibiting chronic diseases. Furthermore, we decided to treat the EGDT group by an algorithm in accordance to Gaieski and colleagues who used the CVP for guiding fluid therapy^10^. However, there are several confounding factors related to the predictive power of CVP. Thus, for example, CVP could be elevated in presence of cardiac tamponade, pulmonary embolism and myocardial infarction^6^. In our study we could rule out the influence of these confounding factors on the CVP by echocardiography. But, both the availably and examiners experience of echocardiography differs among various institutions. Finally, due to the exploratory character of the presented study and the high drop-out rates in both groups, this study might be under-powered.

## Conclusion

In summary, the results of our study demonstrate the feasibility of an early goal directed hemodynamic optimization after cardiac arrest. EGDT is associated with a significant decrease of lactate levels to nearly baseline and is able to significantly improve systolic myocardial function. Additionally, the EGDT group shows a significantly reduced total amount of epinephrine. Although the results of our study suggest that implementation of an EGDT algorithm for post cardiac arrest care seems possible, the impact of algorithms with different hemodynamic parameters should be further investigated within clinical trials that are urgently needed.

## Supplementary Information

Supplementary Information.
