# Supplementary material for: Feasibility and beneficial effects of an early goal directed therapy after cardiac arrest: evaluation by conductance method
Source: Sci Rep. 2021 Mar 5;11:5326. doi: 10.1038/s41598-021-83925-3 (PMC7935910; doi:10.1038/s41598-021-83925-3)
Supplement: Supplementary file 1 — Supplementary Information. [file 41598_2021_83925_MOESM1_ESM.docx]

**Feasibility and beneficial effects of an early goal directed therapy**

**after cardiac arrest: evaluation by conductance method**

Ole Broch^1,2^, MD, Lars Hummitzsch^1^, MD, Jochen Renner^1^, MD, Patrick Meybohm^4^, MD, Martin Albrecht^1^, PhD, Peter Rosenthal^2^, MD, Ann-Christine Rosenthal^2^, MD, Markus Steinfath^1^, MD, Berthold Bein^5^, MD, Matthias Gruenewald^1^, MD

**Running Head:** Early goal directed therapy after cardiac arrest

^1^ Department of Anesthesiology and Intensive Care Medicine, University Hospital Schleswig-Holstein, Campus Kiel, Germany

^2^ Department of Anesthesiology and Intensive Care Medicine, Elbe Hospital Stade, Germany

^3^ Christian-Albrechts-University Kiel, Germany

^4^ Department of Anesthesiology, University Hospital Wurzburg, Germany

^5^ Department of Anesthesiology and Intensive Care Medicine, Asklepios Hospital St. Georg, Hamburg, Germany

OB, ole.broch@elbekliniken.de; LH, [lars.hummitzsch@uksh.de](mailto:lars.hummitzsch@uksh.de); JR, jochen.renner@helios-gesundheit.de; PM, [meybohm_p@ukw.de](mailto:meybohm_p@ukw.de); MA, [martin.albrecht@uksh.de](mailto:martin.albrecht@uksh.de); PR, [peter.rosenthal@uksh.de](mailto:peter.rosenthal@uksh.de); ACR, [ann-christine.rosenthal@uksh.de](mailto:ann-christine.rosenthal@uksh.de); MS, [markus.steinfath@uksh.de](mailto:markus.steinfath@uksh.de); BB, [b.bein@asklepios.com](mailto:b.bein@asklepios.com); MG, [matthias.gruenewald@uksh.de](mailto:matthias.gruenewald@uksh.de)

Correspondence to Lars Hummitzsch, MD, Department of Anesthesiology and Intensive Care Medicine, University Hospital Schleswig-Holstein, Campus Kiel, Schwanenweg 21, D-24105 Kiel, Germany, phone +49 431-500-20701, Fax +49 431-500‑20704, e‑mail: [lars.hummitzsch@uksh.de](mailto:lars.hummitzsch@uksh.de)

**Supplementary material:**

|  | EGDT | noEGDT | **vital threatening thresholds** |
| --- | --- | --- | --- |
| MAP(mmHg) | ≥ 80% BL | 55% - 120% BL | < 40 % BL |
| CVP (mmHg) | ≥ 7 | 2 – 8 | no |
| EF (%) | ≥ 60 | 40 – 70 | no |
| S_cv_O_2_ (%) | ≥ 70 | 50 – 80 | < 45 |

Supplement 1: Thresholds of target variables in both groups. MAP, mean arterial pressure; CVP, central venous pressure; EF, ejection fraction; ScvO_2_, central venous oxygen saturation

**Supplementary material:**


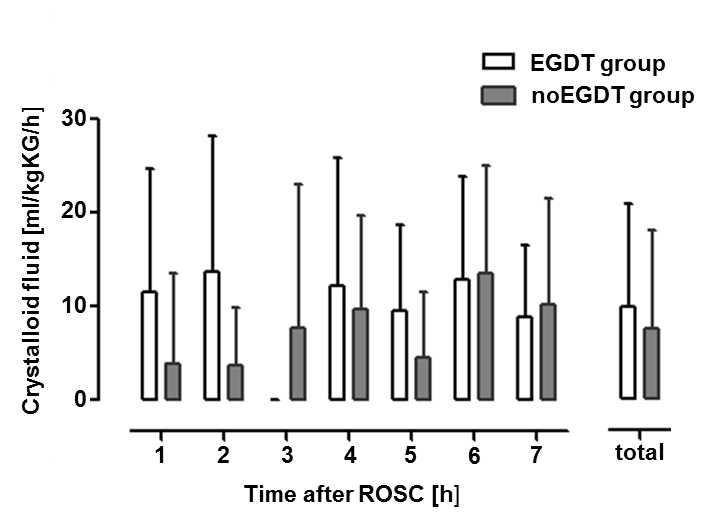


Supplement 2: Sequential and total use of administered fluids within therapeutic algorithm
